# The *fickle* Mutation of a Cytoplasmic Tyrosine Kinase Effects Sensitization but not Dishabituation in *Drosophila Melanogaster*

**DOI:** 10.1080/01677060701249488

**Published:** 2007-10-15

**Authors:** Zoltan Asztalos, Kotaro Baba, Daisuke Yamamoto, Tim Tully

**Affiliations:** a Cold Spring Harbor Laboratory, Cold Spring Harbor, New York, USA; b JST, ERATO, Yamamoto Behavior Genes Project at Mitsubishi-Kasei Institute of Life Sciences, Minami-Ooya, Machida-shi, Tokyo, Japan

**Keywords:** Behavioral, *Drosophila*, Enzymatic mutant, Olfactory jump reflex, Sensitization

## Abstract

*fickle* is a P-element mutation identified from a screen for defects in courtship behavior and disrupts the fly homolog of Bruton's tyrosine kinase (*Btk*) gene (Baba et al., 1999). Here, we show that habituation of the olfactory jump reflex also is defective in *fickle*. Unlike, the prototypical memory mutants, *rutabaga* and *dunce*, which habituate more slowly than normal, *fickle* flies habituate faster than normal. *fickle's* faster-than-normal response decrement did not appear to be due to sensorimotor fatigue, and dishabituation of the jump response was normal. Based on a long-standing “two opponent process” theory of habituation, these data suggested that behavioral sensitization might be defective in *fickle*. To test this hypothesis, we designed a olfactory sensitization procedure, using the same stimuli to habituate (odor) and dishabituate (vortexing) flies. Mutant flies failed to show any sensitization with this procedure. Our study reveals a “genetic dissection” of sensitization and dishabituation and, for the first time, provides a biological confirmation of the two opponent process theory of habituation.

## INTRODUCTION

The *fickle* mutation was isolated from a P element insertional mutagenesis for defective mating behavior and disrupts expression of *Btk29A*, the fly homolog of Bruton's tyrosine kinase (Baba et al., 1999). *Btk* shows extensive amino acid similarities and other *Itk* (*interleukin 2-inducible T-cell kinase*), members of a family of tyrosine kinases that have been shown to be involved in various aspects of neuronal and behavioral plasticity (Grant et al., 1992; Levine et al., 1995; Motro et al., 1996; O'Dell et al., 1991; Rosenblum et al., 1995). This prompted us to evaluate *fickle* for deficits in habituation of the olfactory jump reflex, a well-characterized form of nonassociative learning (Asztalos et al., 2007).

To habituate the olfactory jump response, a single fly is exposed repeatedly to an odor stimulus. Initially, flies show a rigorous jump response to the odor cue, but this response wanes over trials. To distinguish whether this decremental effect results from neural plasticity or from sensorimotor fatigue, flies are dishabituated. Exposure to a single, noxious stimulus (vortexing) produces an immediate reinstatement of high levels of jump response to an odor cue (Asztalos et al., 2007), a result generally observed for central habituation process, but not for fatigue.

Dishabituation might occur either by “erasing” habituation or by sensitizing an already decremented response. The latter notion would predict that sensitization (increased responsiveness to stimuli in naïve animals following a strong, noxious stimulus) and dishabituation (increased responsiveness to the habituating stimulus in decremented animals following a strong, noxious stimulus) share the same molecular mechanism(s). Some evidence suggest the contrary. In *Aplysia* sensitization and dishabituation appear at distinct developmental stages (Marcus et al., 1988). In leech, lesion of S-interneurons disrupts sensitization completely but does not fully eliminate dishabituation (Sahley et al., 1994). Molecular cAMP signaling has been implicated for both sensitization and dishabituation, but PKC modulations affect only dishabituation (Braha et al., 1990; Hochner et al., 1986; Sacktor & Schwartz, 1990). In this study, we show that a *fickle* mutation disrupts sensitization by not dishabituating of the olfactory jump reflex, providing a “genetic dissection” of these two processes. Moreover, *Btk* now is an entry point for future studies to reveal the molecular mechanisms underlying sensitization of the olfactory jump response.

### Materials and Methods

Flies were reared at 25°C and 50% relative humidity either in 16=8 or 11=13 light/dark cycle. *fickle*^*P*^ is a homozygous-viable, hypomorphic mutation produced by insertion of the Bmδ-w P element into an intron of the essential *Btk29A* gene (Baba et al., 1999). The genetic background of the *fickle^P^* stock was “cantonized” by crossing homozygous males to *w*^1118^ *(CS10)* females (cf. Dura et al., 1993) and then, for seven generations, selecting heterozygous females (using the mini-*white*^+^ eye-color marker within Bmδ-w) and mating them to *w*^1118^ *(CS10)* male; this resulted in a true-breeding stock in which each male and female is homozygous for *fickle^P^* (hereafter called *fickle*) was established. For behavioral assays, flies were collected under mild CO2 anesthesia within 24 hs of eclosion and then were tested after another two days. Behavioral experiments were run in an environmentally controlled room at 25°C and 70–80% relative humidity. Highest purity benzaldehyde was from FLUKA and heavy mineral oil was from Fisher Scientific.

### Habituation of the Olfactory Jump Reflex

Habituation was measured according to Asztalos et al. (Asztalos et al., 2007). During training, single males were housed in transparent plastic test tubes. The test tubes were inserted into a lucite base with small holes in it. A vacuum source was connected to the base creating a continuous airflow (1000 ml/min). Odorant was delivered by a computer-controlled 3-way solenoid valve, which switched the air current from air bubbled through mineral oil to air bubbled through a 5% solution of benzaldehyde (BA) in mineral oil. Each training trial consisted of a 4-s odor presentation with intertrial interval (ITI) of 1 or 5 min. A response was recorded if the fly jumped during the 4-s odor presentation or in the following 3 s and was deemed to have habituated when it failed to jump in four consecutive trials (no-jump criterion). Habituation was scored as the number of trials required to reach the no-jump criterion (trials to criterion; TTC).

#### Dishabituation of the Jump Reflex

When a fly reached the no-jump criterion, the chamber was removed from the apparatus and subjected to 75 s of vortexing. Within 15 s, the chamber then was returned to the apparatus, and the fly was exposed to a single odor stimulus during a test-trial. Thus, the dishabituation test occurred 2 min after the last training trial. A Dishabituation Score (DIS) was expressed as the percentage of flies that jumped during the test-trial.

#### Spontaneous Recovery of the Jump Reflex

When a fly reached the no-jump criterion, they were left undisturbed for 2 min, at which time they were exposed to a single odor stimulus during a test-trial. A Spontaneous Recovery Score (SR) was expressed as the percentage of flies that jumped during the test-trial.

#### Initial Jump Response

During habituation and sensitization experiments, we also calculated the percentage of flies that jumped to the first odor presentation. For habituation experiments, we used a relatively high concentration of BA (5%), thereby allowing a response decrement from initially maximal levels of the jump response. For sensitization experiments, we used a relatively low concentration of BA (0.5%), thereby allowing us to observe an increase in jump response from lower initial levels. Initial jump levels also were used to evaluate olfactory acuity and motility of flies.

#### Sensitization of the Olfactory Jump Reflex

To induce sensitization, naïve flies were placed in the training chamber and then immediately were vortexed for 75 s. Within 15 s, vortexed flies were exposed to a test-trial using 0.5% BA. Control flies were placed in the training chamber and left undisturbed for 2 min until the test trial. A Sensitization Score was expressed as the percentage of flies that jumped during the test-trial.

### Data Analysis

The data were analyzed with JMP 3.1 statistical software (SAS Institute Inc.).

*Habituation Score*: Habituation Scores (TTC) were not normally distributed, so we employed *non-parametric Wilcoxon*/*Kruskal-Wallis Tests* (α = 0.05).

*Initial Jump Score*: All the Initial Jump data of a given group (*fickle* or Canton-S flies from experiments using either a 1-min or a 5-min ITI) were pooled together and shuffled randomly to 6 sets of 6 responses, from which 6 “mean percent responses” were computed. In accord with the central limit theorem, the distribution of these means proved to be normal. Means ± SEMs of these sets then were calculated, and groups were compared using Student's t-test.

*Spontaneous Recovery, Dishabituation, and Sensitization Scores*: Mean percentage of response was calculated from a each day of experiment. The Means ± SEMs of these means then were calculated across replicate days. As such, these data were distributed normally, and Student's t-test was used to compare groups.

## RESULTS

### Habituation of the fickle Mutant is Significantly Faster than Normal

**Figure 1:**
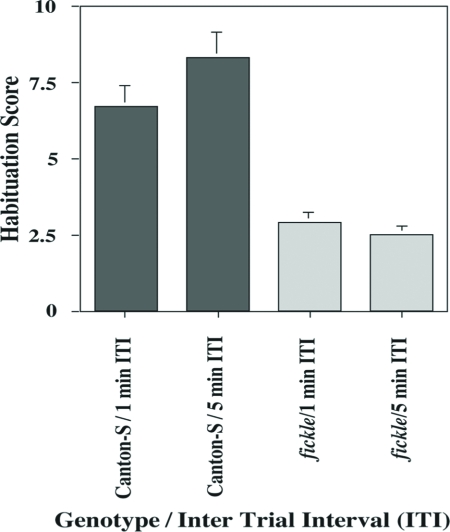
Habituation of the olfactory jump reflex is defective in the *fickle* mutant. Wild-type Canton-S (Can-S, gray bar) and *fickle* (light gray bar) flies were subjected to the olfactory jump reflex habituation assay (see MATERIALS AND METHODS). The mean (±SEM) Habituation Scores for mutants were significantly lower than those for wild-type flies after training with either a 1-min (*p* < 0.001; n = 59 and 73 for *fickle* and Can-S, respectively) or a 5-min ITI (*p* < 0.001; N = 61 and 59 for *fickle* and Can-S, respectively).

**Figure 2:**
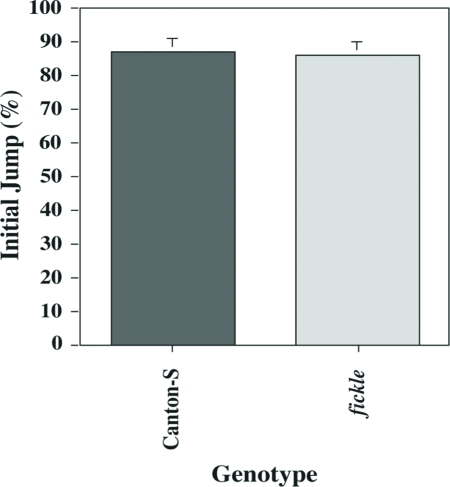
The initial jump response is normal in *fickle*. The means (±SEM) of daily percentage jump response (to 5% BA) on the first trial of the habituation assay are plotted for wild-type Canton-S (gray bar) and *fickle* (light gray bar) flies. The Initial Jump Score of *fickle* individuals (n = 139) was not significantly different from that of wild-type flies (n = 153).

Homozygous *fickle* mutant individuals displayed a more rapid response decrement during the habituation procedure, after training with either a 1-min and 5-min ITI, TTC scores for mutants were significantly lower than those for wild-type flies (Figure 1). Importantly, the initial jump score for *fickle* flies is not significantly different from that for wild-type flies (Figure 2). This observation suggests that mutant flies can smell BA and react to it normally.

### Dishabituation of the fickle Mutant is Normal

**Figure 3:**
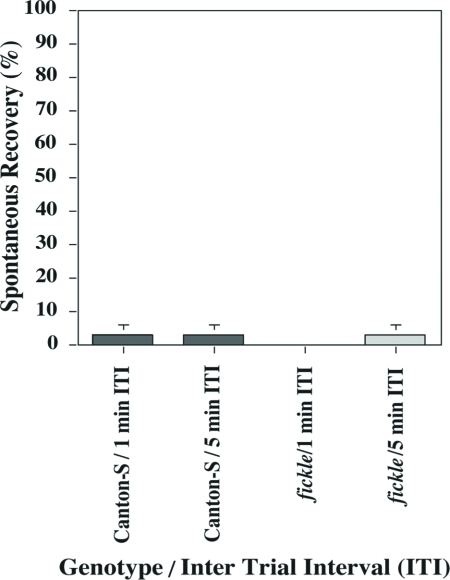
Spontaneous Recovery of the olfactory jump reflex is normal in *fickle*. After each fly reached the no-jump criterion during habituation training, it remained undisturbed in the training chamber for two min, at which time it was subjected to an odor test-trial. Mean Spontaneous Recovery scores (daily percentage jump responses) did not differ (AD-LSD = −0.034, α = 0.05) among the four groups tested [wild-type Canton-S (gray bar) and *fickle* (light gray bar) flies trained with either a 1-min or a 5-min ITI]. n = 60 and 67 flies for *fickle* and Can-S, respectively.

**Figure 4:**
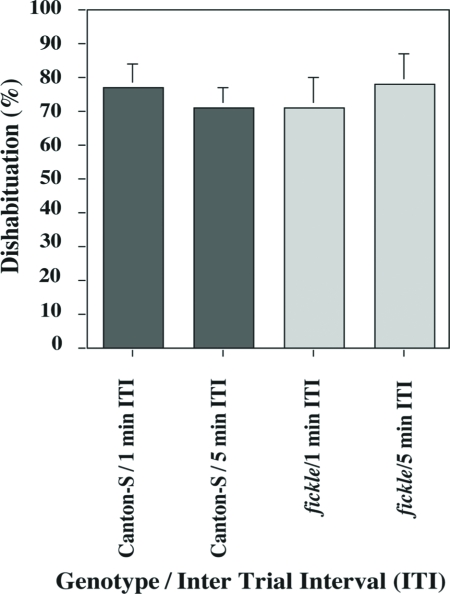
Dishabituation of the olfactory jump reflex is normal in *fickle*. After each fly reached the no-jump criterion during habituation training, its training chamber was removed from the habituation apparatus and vortexed for 75 s. The training chamber then was returned to the apparatus, and the fly was subjected to an odor test-trial 15 s after vortexing. Mean Spontaneous Recovery scores (daily percentage jump responses) did not differ (AD-LSD = −0.005, α = 0.05) among the four groups tested [wild-type Canton-S (gray bar) and *fickle* (light gray bar) flies trained with either a 1-min or a 5-min ITI]. n = 57 and 67 flies for *fickle* and Can-S, respectively.

Once habituated, some flies were left undisturbed in the training chamber for two min and then were subjected to an odor test-trial to assess spontaneous recovery (memory) of the level of jump response. Mean SR scores of mutant flies did not differ significantly from those of wild-type flies after training with either a 1-min or a 5-min ITI (Figure 3). Other groups of flies were dishabituated by vortexing them for 75 s and then subjecting them to an odor test-trial two min after reaching the no-jump criterion. In contrast to the low levels of jump response observed for 2 min spontaneous recovery, jump response levels after the dishabituating stimulus (vortexing) were quite high, almost as high as initial jump levels (see Figure 2), and did not differ between mutant and wild-type flies after training with either a 1-min or a 5-min ITI (Figure 4). Such normal dishabituation also constitutes further proof that sensorimotor responses are normal in mutant flies (see above and Asztalos et al., 2007).

### Sensitization is Blocked in the *fickle* Mutant

**Figure 5:**
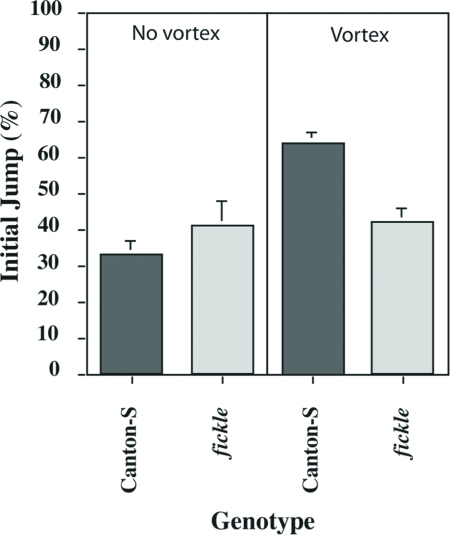
Sensitization of the olfactory jump reflex is defective in *fickle*. A naïve fly either remained undisturbed in the training chamber of the habituation apparatus (No vortex) or was placed in the training chamber, immediately vortexed for 75 s and then placed into the apparatus. In both cases, the fly was subjected to an odor test-trial (0.5% BA) two min after being placed in the training chamber. In the absence of vortexing, Mean Initial Jump Scores did not differ significantly (AD-LSD = −0.16, α = 0.05) between wild-type Canton-S (gray bar) and *fickle* (light gray bar) flies (n = 66 and 66 for *fickle* and Can-S, respectively). The mean Initial Jump Score for wild-type flies after vortexing (n = 66) was significantly higher (AD-LSD = 0.18, α = 0.05) than that without vortexing, while no such increase was detected (AD-LSD = −0.22, α = 0.05) after vortexing (n = 66) in flies homozygous for *fickle*.

Because sensorimotor responses appeared normal in *fickle*, we hypothesized that the faster-than-normal response decrement during the habituation procedure might result from abnormal sensitization (see DISCUSSION). To test this notion, we designed a sensitization procedure with the same stimuli used for the habituation (BA) and dishabituating (vortexing) procedures (see MATERIALS AND METHODS). To measure such sensitization-induced increases in the olfactory jump reflex, we had to reduce the concentration of BA used—from 5% to 0.5%—to yield initial jump response levels around 40% (Figure 5). At this lower BA concentration, we again observed no statistically significant difference between *fickle* flies and wild-type ones. When queried with an odor test-trial 15 s after vortexing, the mean jump response level for wild-type flies was significantly higher than that with no vortexing (Figure 5), and this sensitized response decayed away within 60 s (data not shown). In contrast, vortexing yielded no significant increase in the mean jump response of flies homozygous for the *fickle* mutation (Figure 5).

## DISCUSSION

In this report, we show that the *fickle* mutant exhibits faster-than-normal habituation (Figure 1) and defective sensitization (Figure 5) of the olfactory jump reflex. Significantly, initial jump response levels do not differ between mutant and wild-type flies after both higher (Figure 2) and lower (Figure 5) odor concentrations, arguing that basal sensorimotor components of the jump reflex are normal in mutant flies. Further support for this conclusion derives from dishabituation experiments, wherein mutant flies again performed normally (Figure 4). The occurrence of strong dishabituation rules out the possibility that (i) the observed response decrement during the habituation procedure or (ii) the minimal spontaneous recovery (memory retention) within two minutes after training (Figure 3), are the result of sensorimotor fatigue from repeated trials during training (cf. Asztalos et al., 2007).

So, how then might we explain the faster-than-normal habituation displayed by *fickle*? Past studies of other animals have yielded a “two opponent process theory” of habituation (Groves and Thompson, 1970). This theory postulates that a stimulus initially induces a sensitization process, and then repeated presentations of that stimulus induce a habituation process that diminishes or “opposes” sensitization. The observed response decrement during a habituation procedure then reflects the net effect of these two opposing neural processes. With this model in mind, we hypothesized that the faster-than-normal response decrement observed in the *fickle* mutant might occur because of a defect in sensitization. We confirmed this hypothesis (Figure 5) by developing a novel assay for sensitization to odor cues after a strong mechanical stimulus (vortexing).

To keep behavioral responses comparable, we designed a sensitization assay using the same stimuli as those used for the habituation (BA) and dishabituation (vortexing). Consequently, the observation that sensitization but not dishabituation is defective in *fickle* unequivocally differentiates the cellular and/or molecular mechanisms underlying these two behavioral phenomena. This finding is in general agreement with results from electro-physiological experiments in *Aplysia* (Braha et al., 1990; Hochner et al., 1986; Marcus et al., 1988; Sacktor & Schwartz, 1990) and cell ablation experiments in leech (Sahley et al., 1994), suggesting both biochemical and anatomical differences between sensitization and dishabituation.

These data suggest a role for *Btk* in *Drosophila* behavioral plasticity, which extends similar observations from other model systems (Grant et al., 1992; Levine et al., 1995; Motro et al., 1996; O'Dell et al., 1991; Rosenblum et al., 1995). Other recent work in *Drosophila* begins to hint at additional components of a possibly novel signaling pathway. Behavioral screens for mutants in olfactory long-term memory have identified mutations in *src64B* (Dubnau et al., 2003 and see Nicolai et al., 2003) and *PTP10D* (Qian et al., 2007), that latter of which interacts with *gp150* (Qian et al., 2007) which itself is know to regulate *Notch* (Ge et al., 2004). Thus, molecular mechanisms of behavioral plasticity in *Drosophila* slowly are being extended beyond the canonical cAMP signaling pathway (Margulies et al., 2005). Further studies on *fickle* are needed, however, (i) to discern its developmental from physiological effects and (ii) to determine to what extent *Btk* functions during other types of (associative) learning and memory.
